# Supplementary material for: A Critical View of the Application of the APEX Software (Aqueous Photochemistry of Environmentally-Occurring Xenobiotics) to Predict Photoreaction Kinetics in Surface Freshwaters
Source: Molecules. 2019 Dec 18;25(1):9. doi: 10.3390/molecules25010009 (PMC7017383; doi:10.3390/molecules25010009)
Supplement: Supplementary file 1 [file molecules-25-00009-s001.zip › 3.2.4_gcc-4.4.0.zip!/3.2.4_gcc-4.4.0/3.2.4_gcc-4.4.0/doc/octave/html/interpreter/Explicit-and-Implicit-Conversions.html]

Explicit and Implicit Conversions - Untitled


Previous: Creating Permutation Matrices,
Up: Basic Usage


---

#### 20.1.3 Explicit and Implicit Conversions

The diagonal and permutation matrices are special objects in their own right. A number
of operations and built-in functions are defined for these matrices to use special,
more efficient code than would be used for a full matrix in the same place. Examples
are given in further sections.

To facilitate smooth mixing with full matrices, backward compatibility, and
compatibility with matlab, the diagonal and permutation matrices should allow
any operation that works on full matrices, and will either treat it specially,
or implicitly convert themselves to full matrices.

Instances include matrix indexing, except for extracting a single element or
a leading submatrix, indexed assignment, or applying most mapper functions,
such as exp.

An explicit conversion to a full matrix can be requested using the built-in
function full. It should also be noted that the diagonal and permutation
matrix objects will cache the result of the conversion after it is first
requested (explicitly or implicitly), so that subsequent conversions will
be very cheap.
